# Effects of Simulated Microgravity on *Limnospira platensis* Growth and Transcript Expression Related to Photosystem and Open Reading Frames

**DOI:** 10.3390/cimb48101061

**Published:** 2026-10-14

**Authors:** Trang Thi Huyen Nguyen, Thuy Thi Kim Dang, Giap Dang Do, Tuan Trong Tran, Chi Nguyen Quynh Ho, Quan Minh To, Mai Thi Phuong Nguyen, Cuong Phan Minh Le, Anh Phan Nguyet Do, Linh Thi Anh Dinh, Nhan Lu Chinh Phan, Chung Chinh Doan, Han Thai Minh Nguyen, Long Thanh Le

**Affiliations:** 1 Institute of Life Sciences, Vietnam Academy of Science and Technology, Ho Chi Minh City 700000, Vietnam; nthtrang@ils.vast.vn (T.T.H.N.); dtkthuy@ils.vast.vn (T.T.K.D.); ddgiap@ils.vast.vn (G.D.D.); tttuan@ils.vast.vn (T.T.T.); hnqchi@ils.vast.vn (C.N.Q.H.); ntpmai@ils.vast.vn (M.T.P.N.); lpmcuong@ils.vast.vn (C.P.M.L.); dpnguyetanh1705@gmail.com (A.P.N.D.); micadinh34@gmail.com (L.T.A.D.); plcnhan@ils.vast.vn (N.L.C.P.); dcchung@ils.vast.vn (C.C.D.); 2 Faculty of Biology, Graduate University of Science and Technology, Hanoi 10000, Vietnam; 3 Faculty of Biology and Biotechnology, Ho Chi Minh City University of Science, Ho Chi Minh City 700000, Vietnam; tomquan@hcmus.edu.vn; 4 Biotechnology Innovation Center, University of New Hampshire, Manchester, NH 03101, USA; han.nguyen@unh.edu

**Keywords:** *Limnospira platensis*, *Arthrospira platensis*, simulated microgravity, three-dimensional clinostat, photosystem gene expression, open reading frame, bioregenerative life-support system

## Abstract

*Limnospira platensis* (formerly *Arthrospira platensis*) was cultured for 14 days under simulated microgravity (SMG) generated by a three-dimensional clinostat and compared with motion-matched controls using optical density, live/dead staining and real-time RT-PCR. Control cultures appeared to proliferate faster initially but declined after day 8 and died by day 14, whereas SMG cultures maintained a prolonged exponential phase and reached a final OD_750_ of 1.43 ± 0.05, approximately 29% above the control maximum (1.11 ± 0.12; *p* = 0.040). Specific growth rates did not differ over days 0–6 (*p* = 0.13), but between days 8 and 12, the control declined (−0.111 ± 0.008 day^−1^) while SMG cultures continued to grow (0.025 ± 0.011 day^−1^; *p* < 0.000001). Live/dead staining confirmed that SMG-cultured cells retained normal pigmentation and viability throughout, whereas control cells exhibited progressive cell death from day 10 onward. *ftsZ* was significantly lower under SMG on day 2 and higher on day 12. Eleven of 19 photosystem and open reading frame genes were significantly higher in the control on day 2, in SMG on day 10 and in the control again on day 12. At matched cell density (control day 6 versus SMG day 10; OD_750_ *p* = 0.52), 19 of the 20 genes remained significantly higher under SMG, so this divergence is not an artefact of the growth stage. Reducing mechanical shear stress through SMG thus maintains physiological integrity, prolongs the productive growth phase and enhances biomass accumulation, supporting the use of *L. platensis* in bioregenerative life-support systems.

## 1. Introduction

Sustained human presence beyond low Earth orbit depends on bioregenerative life-support systems (BLSSs) that recycle waste into oxygen, water and food [1], and the filamentous cyanobacterium *Limnospira platensis* is a leading candidate organism [2]. Formerly described as *Spirulina platensis* and *Arthrospira platensis*, the commercially grown taxa were transferred to the new genus *Limnospira* [3] and later consolidated into the single species *L. platensis* [4] (NCBI Taxonomy ID 118562 [5]); this name is used throughout, while names from earlier studies are reproduced as published. *L. platensis* combines rapid autotrophic growth, efficient conversion of CO_2_ into oxygen and edible biomass rich in protein, B-vitamins, antioxidants and all essential amino acids [2,6,7]; its thin cell wall (86% digestible polysaccharides) and Generally Recognized As Safe (GRAS) status [2,7] make it simultaneously a food, an air-revitalization agent and a CO_2_ sink [8,9]. In the Micro-Ecological Life Support System Alternative (MELiSSA) programme of the European Space Agency (ESA), interconnected bioreactors regenerate air, water and biomass [10,11], and a photobioreactor colonized by axenic *Limnospira indica* PCC 8005 produces oxygen and biomass from crew CO_2_ and nitrate [12,13]. The ARTEMISS and ARTHROSPIRA experiments operated such a photobioreactor aboard the International Space Station (ISS) from 2018 [13,14], and over an approximately five-week run, microgravity had no first-order effect on its oxygen production rate [14]. Recent hardware sustained *L. indica* for up to nine weeks, with biomass in the expected range but oxygen below prediction [7], and in ground batch studies lower light intensities (36–75 µmol photons m^−2^ s^−1^) yielded higher biomass and pigment content than intensities above 100 µmol photons m^−2^ s^−1^ [15].

The spaceflight response of this organism remains incompletely resolved, partly because true microgravity is difficult to separate from its fluid-mechanical consequences, namely the loss of buoyancy-driven convection and sedimentation and the altered mass transfer around suspended cells [6,16]. Under low-shear simulated microgravity, *L. indica* grew more slowly, downregulating ribosomal proteins and nitrate transporters while upregulating carboxysome and photosystem proteins, changes attributed to elevated oxygen partial pressure and mass-transfer-limited carbon starvation rather than to gravity sensing [6,17,18]. *L. platensis* also survives gamma doses up to 400 kGy [19], yet the combined long-term effects of radiation and microgravity on growth, composition and genetic stability remain unmeasured [2,20,21,22,23]. A dimethyl sulfoxide–trehalose system now preserves NIES-39 at −80 °C for at least a year, removing the historical need for continuous subculture [1,24], and the Space Algae-2 investigation, rehearsed at the HI-SEAS lunar analog, is testing the genetic stability of NIES-39 over six months of ISS culture by multi-omics analysis [2]. The JAXA Space Surface trial and regolith-simulant studies [25] extend the organism’s role towards planetary habitats [26,27], and earlier work on batch growth and stress-driven shifts between protein and carbohydrate [28], protein quality [8], dehydration morphometry [24] and photobioreactor deployment [12,29] frames the question addressed here.

Previous simulated-microgravity and spaceflight studies of *Limnospira* have characterized growth kinetics, pigment content, oxygen production and the proteomic response of the photosynthetic apparatus [6,7,14], but generally at one or a few endpoints. The present study differs in three respects: it samples every two days from inoculation to culture collapse, revealing that the difference between SMG and control cultures changes direction over time rather than reflecting a fixed up- or down-regulation; it relates this time course to the point at which the control culture dies; and it compares transcript abundance at matched cell densities as well as matched times, so that growth-stage effects can be assessed. Simulated microgravity generated by a three-dimensional clinostat was combined with growth measurements, viability staining and real-time RT-PCR to determine how reduced gravitational and mechanical cues affect the growth, survival and transcriptional programme of *L. platensis*, with the practical goal of optimizing its cultivation for space-life support.

## 2. Materials and Methods

### 2.1. Preparation of Culture Medium

*L. platensis* (supplied by Xanh va Xanh Manufacturing and Trading Co., Ltd. (Nha Trang, Vietnam)) was cultivated in Zarrouk medium [30]. The medium was prepared from two 2×-concentrated stock solutions, which were kept separate to prevent precipitation of carbonate and phosphate salts, together with an A5 trace element solution; the complete composition and reagent sources are given in Table S1. The final medium was obtained by combining 500 mL of Stock 1, 500 mL of Stock 2, and 1 mL of A5 solution per litre. The pH was adjusted to 9.0 ± 0.2, and the medium was sterilized by autoclaving at 121 °C for 15 min.

### 2.2. Preparation of the Simulated Microgravity System

Simulated microgravity was generated using a Gravite^®^ gravity controller (Space Bio-Laboratories Co., Ltd., Hiroshima, Japan), a three-dimensional clinostat that continuously reorients the sample by about two independent axes so that the time-averaged gravity vector approaches zero while the cells remain in suspension [16,31]. Mode C (3 rpm), recommended by the manufacturer for cell-based studies, was used throughout. Control cultures were incubated under identical temperature and illumination on a tube rotator mixer that rotated the tube 180° clockwise and back at 3 cycles min^−1^, so that both groups received continuous gentle agitation and comparable gas exchange and differed essentially in whether the gravity vector was randomized. This is a motion-matched control rather than an optimal-growth photobioreactor culture, a distinction considered in the Discussion.

Both groups were cultivated in sterile 5 mL cryopreservation tubes completely filled with culture (initial OD_750_ 0.30), sealed with a Teflon membrane to permit gas exchange and incubated at 25 °C under a cool-white fluorescent lamp (2000 lux at the vessel surface; 12 h light:12 h dark), without headspace exchange. Samples for optical density, light microscopy, viability staining and RNA extraction were withdrawn every two days from day 0 to day 14. Optical density was measured at 750 nm against a medium blank using an SP-300 spectrophotometer (OPTIMA, Tokyo, Japan). Each condition comprised five independent biological replicates for growth measurements and three for transcript analysis.

### 2.3. Growth Rate Evaluation

Specific growth rates (*µ*) were calculated for each replicate as follows:

$$\mu =\frac{\left(\ln X_{1}-\ln X_{2}\right)}{\left(t_{2}-t_{1}\right)}$$

where *X*_1_ and *X*_2_ are the biomass proxies (OD) at the beginning and end of an interval and *t* is time in days, following the conventional approach used for microalgal and cyanobacterial growth kinetics [32].

### 2.4. Viability Evaluation

Live and dead *L. platensis* cells were distinguished using the Viability/Cytotoxicity Assay Kit for Bacteria Live & Dead Cells (30027, Biotium, Fremont, CA, USA), which combines a membrane-permeant green nucleic acid stain with a membrane-impermeant red counterstain, following the manufacturer’s instructions. Fluorescence images were acquired using the Cytell Cell Imaging System (GE Healthcare Life Sciences, Uppsala, Sweden). Cell morphology and trichome organization were examined in parallel under bright-field illumination and assessed with reference to previously described morphometric changes in this organism under stress [24].

### 2.5. Quantitative Real-Time PCR Analysis

Total RNA was extracted from *L. platensis* using the Animal Tissue & Cell RNA Extraction Kit (G3640, Servicebio, Wuhan, China) according to the manufacturer’s protocol, with an additional disruption step in which cell pellets were frozen in liquid nitrogen and ground to a fine powder using a pre-chilled mortar and pestle prior to lysis. RNA concentration and purity were determined using a NanoVue Plus spectrophotometer (28-9569-57, GE Healthcare Life Sciences, Uppsala, Sweden), and RNA integrity was verified by 1% agarose gel electrophoresis. Genomic DNA was removed by treatment with DNase I (G3342, Servicebio, Wuhan, China) according to the manufacturer’s instructions.

Transcript levels were quantified for 20 target genes: the cell-division gene *ftsZ*; four photosystem I genes, *psaA* and *psaB* (P700 chlorophyll *a* apoproteins A1 and A2), *psaC* (iron–sulfur centre subunit) and *psaD* (reaction centre subunit II); four photosystem II genes, *psbA* (D1 protein), *psbB* (CP47 chlorophyll *a*-binding protein), *psbDII* (D2 protein, gene copy II) and *psbE* (cytochrome b_559_ α subunit); and eleven annotated open reading frames (ORFs 1242, 1751, 2155, 3656, 3909, 4133, 4139, 4691, 4792, 4935 and 5692), numbered as in the work of Wang et al. [32]; their protein accession numbers, gene products and functional categories are listed in Table S3. Primers for the ORFs were taken from Huili et al. [33], with modifications indicated in Table S2; primers for *ftsZ*, the *psa* and *psb* genes and the 16S rRNA reference gene were designed against the *Arthrospira platensis* NIES-39 genome (GenBank AP011615) (Table S2). Primers were synthesized by PHUSA Genomics (order number: OLI-PO#0OJ-250612-5, Can Tho, Vietnam).

RT-qPCR was performed on a PikoReal 96 Real-Time PCR System (Thermo Fisher Scientific, Waltham, MA, USA). Each 10 µL reaction contained 5 µL of 2× SensiFAST SYBR No-ROX Mix (Bioline, London, UK), 1 µL of forward and reverse primers, 2 µL of cDNA and 2 µL of nuclease-free water. Cycling comprised 95 °C for 5 min; 40 cycles of 95 °C for 30 s, with primer-specific annealing at 53–61.3 °C (Table S2) for 30 s and 70 °C for 30 s; and melting-curve analysis from 60 °C to 95 °C. Relative abundance was calculated by the 2^−ΔΔCt^ method [34], with 16S rRNA as the reference and day 0 as the calibrator. 16S rRNA is among the most stable RT-qPCR reference genes in cyanobacteria, including *Lyngbya aestuarii* CCY 9616 and *Nostoc* sp. PCC 7120, across growth and stress conditions [35], although its stability under simulated microgravity in *Limnospira* has not been established.

### 2.6. Statistical Analysis

Data are presented as mean ± standard deviation (SD) of five independent biological replicates for growth measurements and three for transcript analysis, unless stated otherwise. Optical density and relative transcript levels were analyzed by two-way analysis of variance (ANOVA) with treatment (control or SMG) and sampling time as factors, followed by Tukey’s multiple comparisons test to compare the control and SMG groups at each sampling time; Tukey’s test was also applied to the density-matched comparisons between different sampling times (Section 3.3). Day 14 was excluded from the ANOVA because the control culture was no longer viable and no paired measurement was available. Specific growth rates (µ) were compared between the control and SMG groups for each interval by multiple unpaired *t*-tests, with *p*-values adjusted for multiple comparisons using the Holm–Šídák method. A *p*-value below 0.05 was considered statistically significant (* *p* < 0.05; ** *p* < 0.01; *** *p* < 0.001; **** *p* < 0.0001); differences that did not reach this threshold are denoted “ns” in the figures. Statistical analyses were performed and graphs were prepared using GraphPad Prism version 10.2.3 (GraphPad Software, San Diego, CA, USA).

## 3. Results

### 3.1. Effects of SMG on L. platensis Proliferation

**Figure 1:**
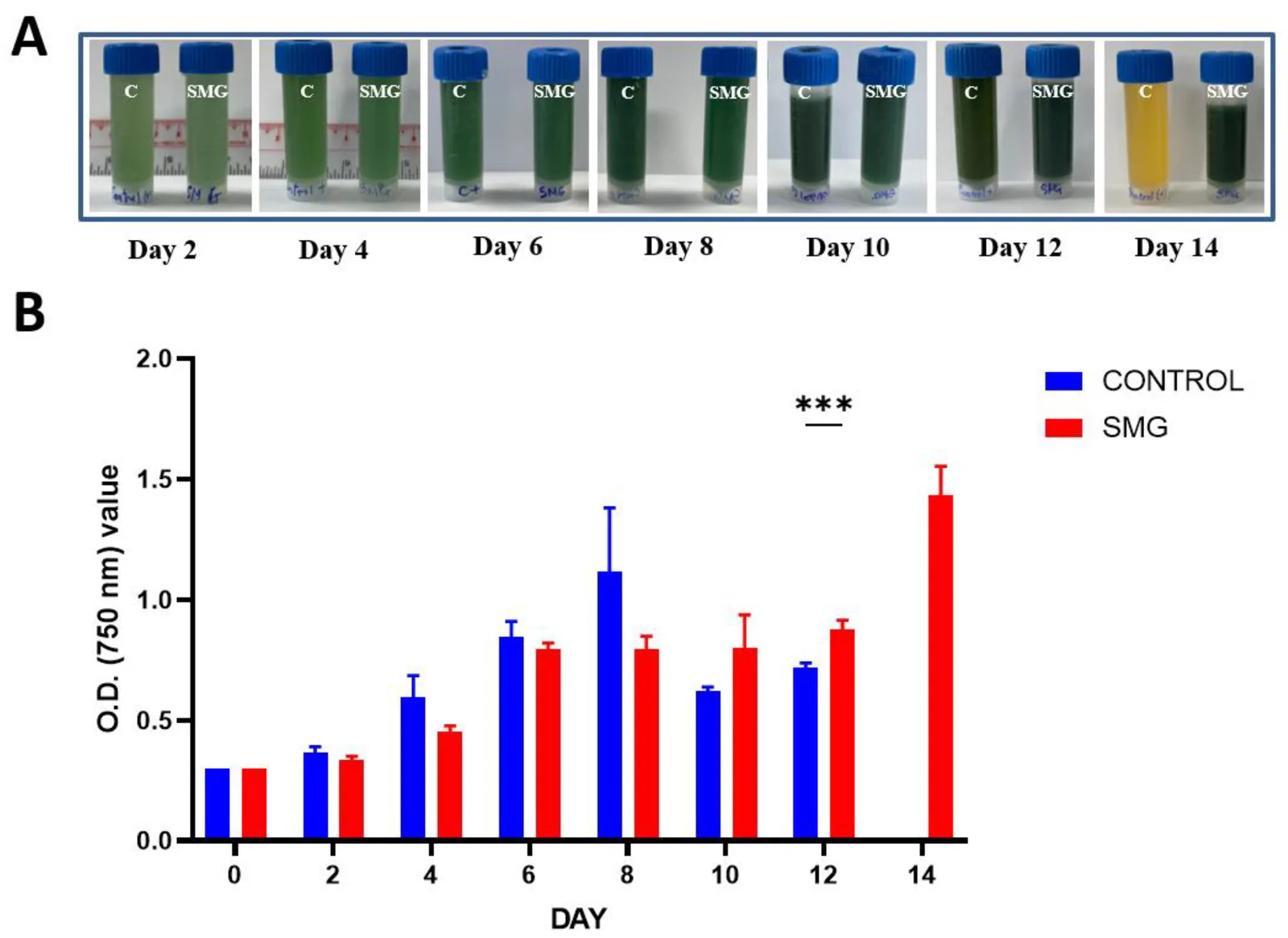
The proliferation of *L. platensis* in the control and SMG groups. (**A**) *L. platensis* cultivated in 5 mL tubes in the control and SMG groups. (**B**) OD_750_ of *L. platensis* from day 0 to day 14. Data are mean ± SD of five independent biological replicates. *** *p* < 0.001, control versus SMG at the same time point. No statistical comparison is shown for day 14 because the control culture was no longer viable at that time point and no paired measurement was available.

Control cultures reached their peak OD_750_ on day 8 (1.11 ± 0.12, versus 0.79 ± 0.03 for SMG), reflecting a faster apparent early growth rate, then declined to 0.62 ± 0.01 on day 10, increased only slightly by day 12 and contained no viable biomass by day 14 (Figure 1). SMG cultures remained at approximately 0.80 between days 6 and 10 and then increased to 0.88 ± 0.02 on day 12 and 1.43 ± 0.05 on day 14; the difference between groups was marked as significant only on day 12 (*p* < 0.001; Figure 1B). SMG therefore did not accelerate initial growth but prolonged the exponential phase and delayed the decline, so that the maximum OD_750_ of the SMG culture exceeded that of the control by 28.7% (*p* = 0.040).

Specific growth rates (Table 1) did not differ over the shared exponential phase (days 0–6: control, 0.172 ± 0.012 day^−1^; SMG, 0.162 ± 0.006 day^−1^; mean ± SD; *p* = 0.13), indicating that the lower early optical density of SMG cultures reflects a modest lag rather than reduced growth capacity. Across each culture’s full exponential phase, µ was 0.162 ± 0.026 day^−1^ for the control (days 0–8) and 0.112 ± 0.006 day^−1^ for SMG (days 0–14; *p* = 0.006), reflecting slower but more sustained growth under SMG. Between days 8 and 12, the control showed net cell loss (−0.111 ± 0.008 day^−1^), whereas the SMG culture continued to accumulate biomass (0.025 ± 0.011 day^−1^; *p* < 0.000001).

**Figure 2:**
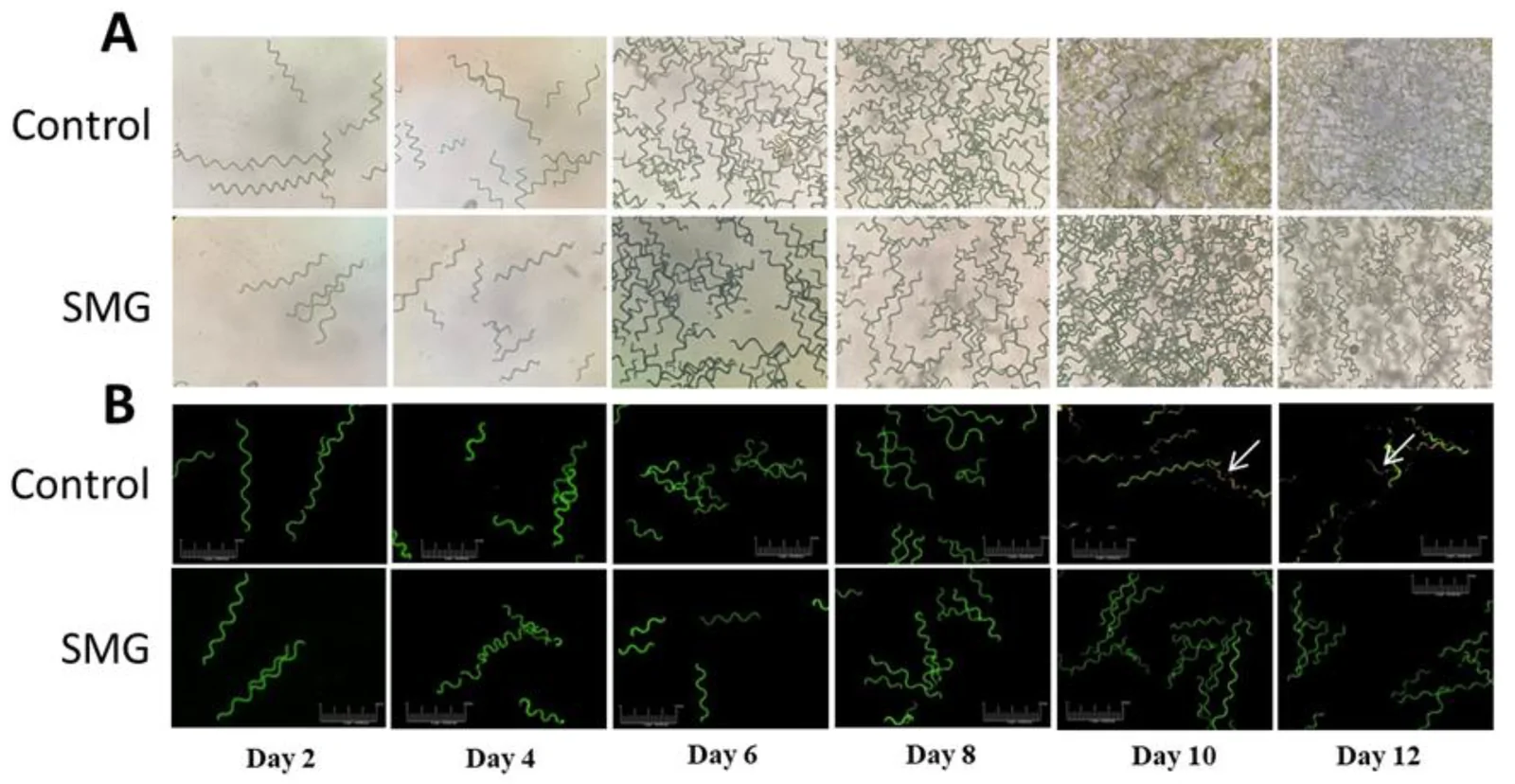
The morphology of *L. platensis* in the control and SMG groups. (**A**) *L. platensis* morphology under bright-field illumination. (**B**) Cell viability determined by fluorescence staining (green, live cells; yellow, dead cells; white, arrows). Scale bars are shown in each panel.

Figure 2A shows the morphology of *L. platensis* in the control and SMG groups. *L. platensis* in the control group maintained its normal color (cytoplasm is green) during the early growth phase (from day 0 to day 8). On days 10 and 12, the dead cells appeared in the *L. platensis* population (cytoplasm is pale yellow). The results of live/dead staining of *L. platensis* cells further support this finding (Figure 2B). Conversely, *L. platensis* in the SMG group maintained normal morphology and color during the culture process from day 0 to day 12.

**Figure 3:**
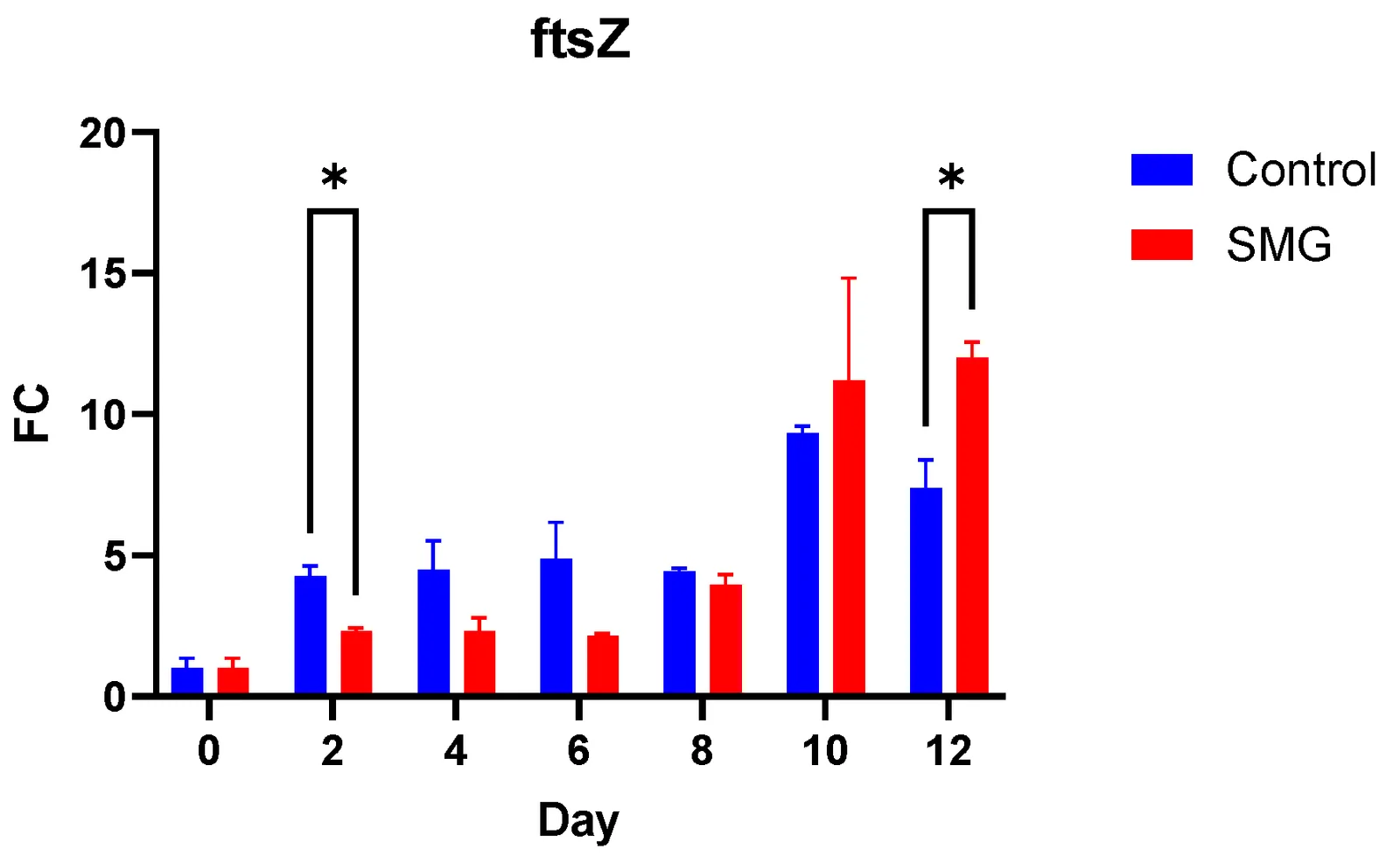
Transcript expression of *ftsZ* in *L. platensis* from day 0 to day 12, expressed as fold change (FC) relative to day 0 and normalized to the 16S rRNA gene. Data are mean ± SD of three independent biological replicates. * *p* < 0.05; control versus SMG at the same time point.

Relative to day 0, *ftsZ* transcript levels increased in both groups (Figure 3). They tended to be higher in the control on days 2–6, differed significantly only on day 2, did not differ significantly on days 4–10, and were significantly higher under SMG on day 12.

### 3.2. Effects of SMG on the Transcript Expression Related to PSI and PSII System

**Figure 4:**
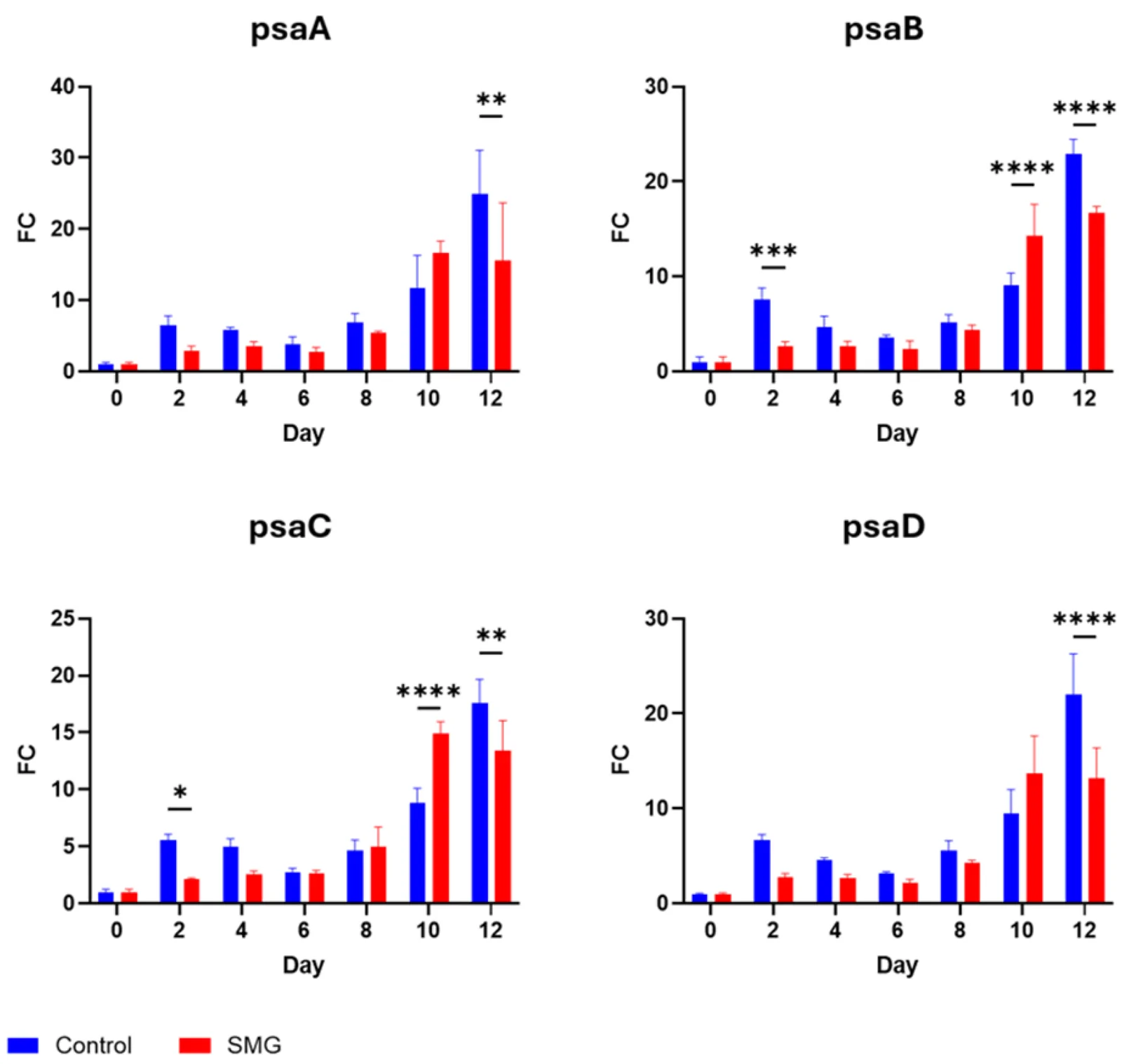
Transcript expression of photosystem I genes in *L. platensis* from day 0 to day 12, expressed as fold change (FC) relative to day 0 and normalized to the 16S rRNA gene. Data are mean ± SD of three independent biological replicates. **** *p* < 0.0001; *** *p* < 0.001; ** *p* < 0.01; * *p* < 0.05, control versus SMG at the same time point.

Fold changes are given below as control versus SMG. Among the photosystem I genes (Figure 4), *psaA* and *psaD* remained stable from day 2 to day 10 and differed only on day 12, when control levels exceeded SMG (*psaA*, C: 24.93 ± 6.16 vs. SMG: 15.60 ± 8.08; *psaD*, C: 22.04 ± 4.24 vs. SMG: 13.23 ± 3.16). For *psaB* and *psaC*, the direction of the difference reversed twice, with both reversals being significant: control exceeded SMG on day 2 (*psaB*, C: 7.57 ± 1.21 vs. SMG: 2.71 ± 0.43; *psaC*, C: 5.55 ± 0.52 vs. SMG: 2.13 ± 0.21), SMG exceeded control on day 10 (*psaB*, C: 9.02 ± 1.34 vs. SMG: 14.32 ± 3.28; *psaC*, C: 8.78 ± 1.32 vs. SMG: 14.95 ± 0.99), and control again exceeded SMG on day 12 (*psaB*, C: 22.93 ± 1.52 vs. SMG: 16.69 ± 0.69; *psaC*, C: 17.55 ± 2.12 vs. SMG: 13.93 ± 2.65), as control expression rose sharply while SMG levels changed little.

**Figure 5:**
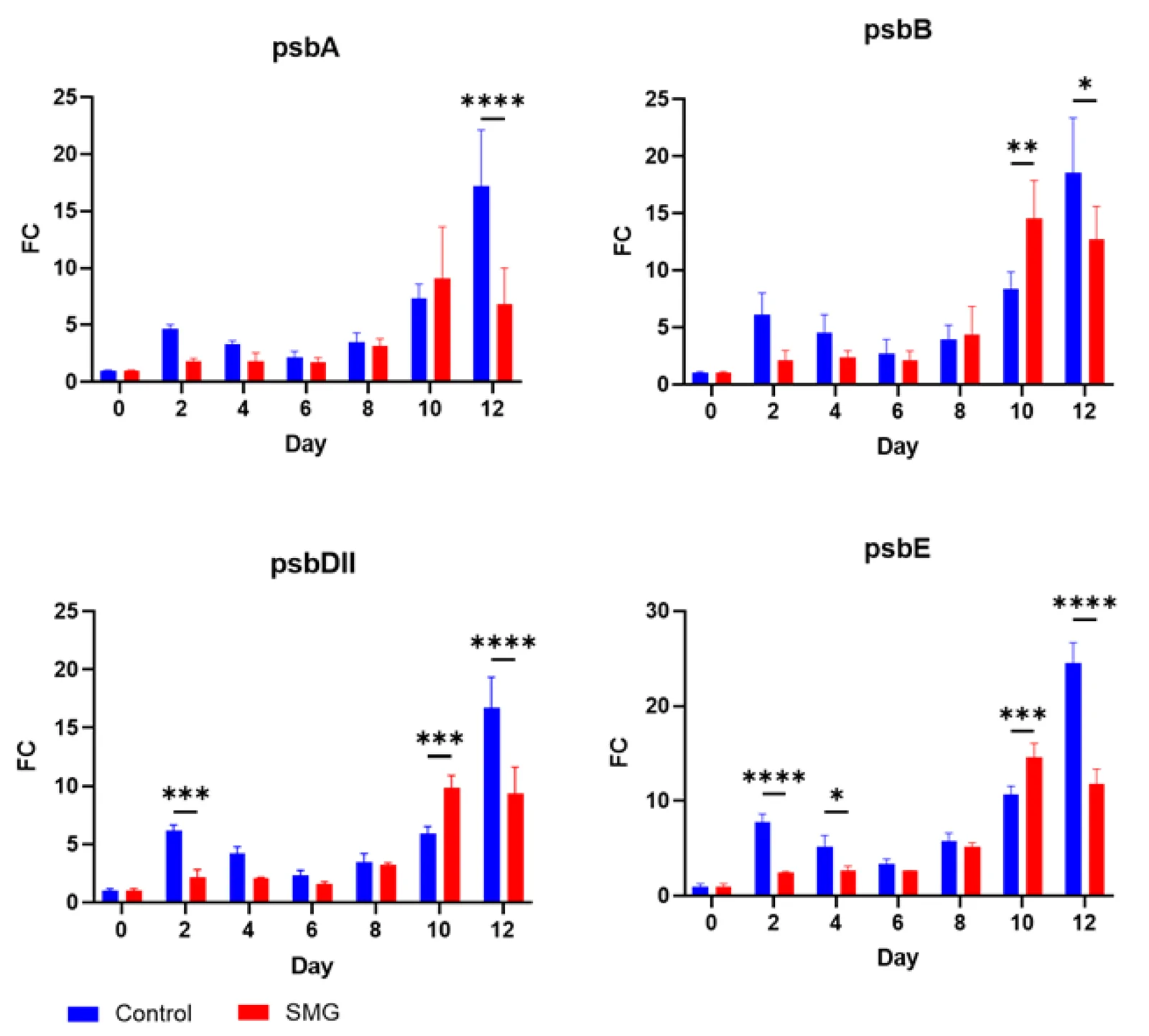
Transcript expression of photosystem II genes in *L. platensis* for the control and SMG groups from day 0 to day 12, expressed as fold change (FC) relative to day 0 and normalized to the 16S rRNA gene. Data are mean ± SD of three independent biological replicates. **** *p* < 0.0001; *** *p* < 0.001; ** *p* < 0.01; * *p* < 0.05, control versus SMG at the same time point.

Among the photosystem II genes (Figure 5), *psbA* remained low in both groups until day 8, tended to be higher in the control on days 2–6 and under SMG on day 10, and differed significantly only on day 12 (C: 17.17 ± 4.97 vs. SMG: 6.86 ± 3.15). *psbB* was non-significantly higher in the control on days 2–6, significantly higher under SMG on day 10 (C: 8.38 ± 1.50 vs. SMG: 14.53 ± 3.35) and higher in the control on day 12 (C: 18.55 ± 4.84 vs. SMG: 12.75 ± 2.87). *psbDII* and *psbE* showed both reversals: control exceeded SMG on day 2 (*psbDII*, C: 6.19 ± 0.46 vs. SMG: 2.13 ± 0.70; *psbE*, C: 7.75 ± 0.87 vs. SMG: 2.45 ± 0.13) and, for *psbE*, on day 4 (C: 5.22 ± 1.52 vs. SMG: 2.64 ± 0.50); SMG exceeded control on day 10 (*psbDII*, C: 5.91 ± 0.63 vs. SMG: 9.85 ± 1.06; *psbE*, C: 10.69 ± 0.87 vs. SMG: 14.55 ± 1.52); and control was markedly higher on day 12 (*psbDII*, C: 16.75 ± 2.60 vs. SMG: 9.37 ± 2.24; *psbE*, C: 24.56 ± 2.11 vs. SMG: 11.85 ± 1.52).

**Figure 6:**
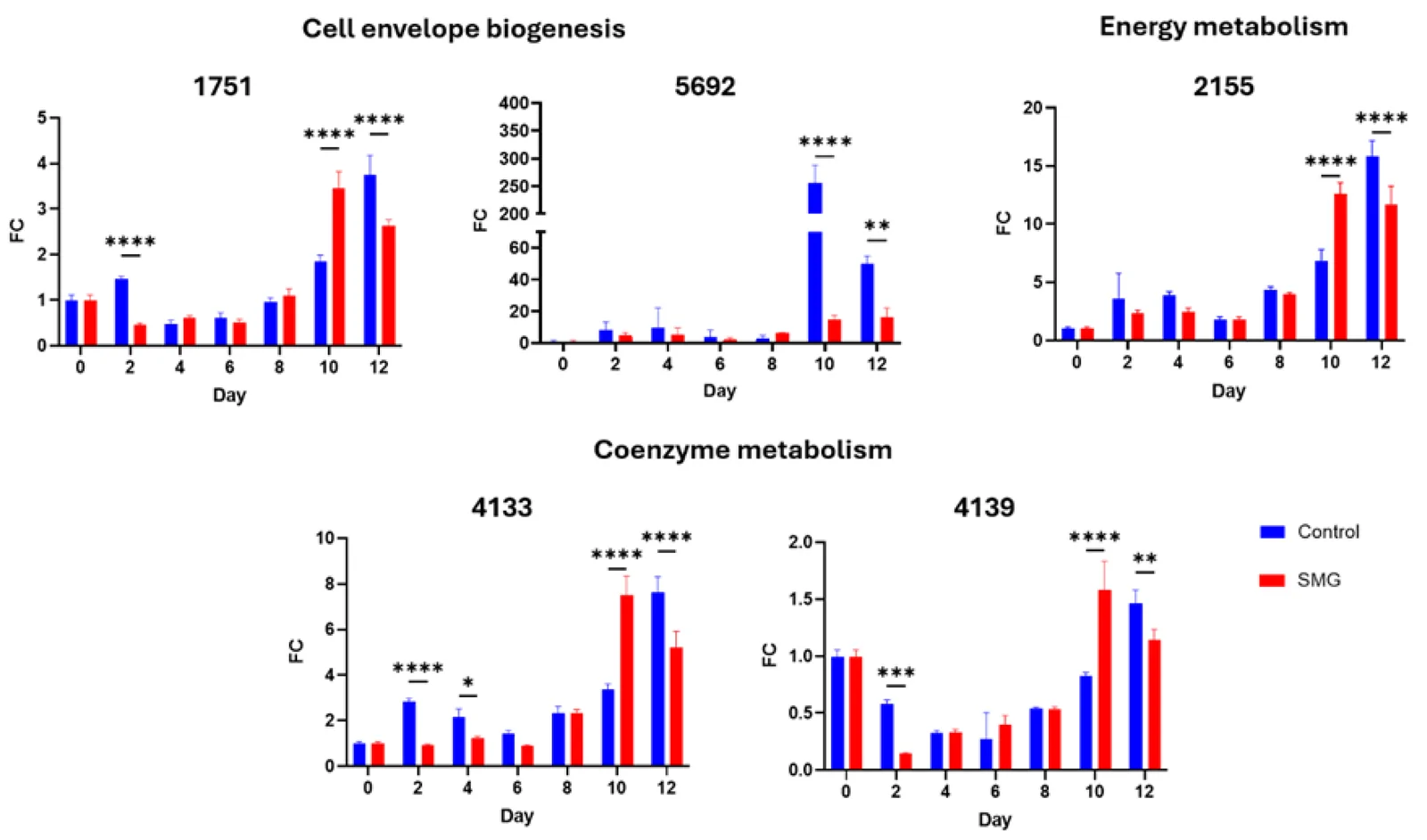
Transcript expression of functional open reading frames associated with cell envelope biogenesis, energy metabolism and coenzyme metabolism in *L. platensis* in the control and SMG groups from day 0 to day 12, expressed as fold change (FC) relative to day 0 and normalized to the 16S rRNA gene. Data are mean ± SD of three independent biological replicates. **** *p* < 0.0001; *** *p* < 0.001; ** *p* < 0.01; * *p* < 0.05; control versus SMG at the same time point.

Among the open reading frames in Figure 6, ORF 1751, ORF 4133 and ORF 4139 showed the same two significant reversals: control exceeded SMG on day 2 (and, for ORF 4133, on day 4) and the groups were otherwise similar until day 8; SMG exceeded control on day 10 (ORF 4133, C: 3.56 ± 0.26 vs. SMG: 7.5 ± 0.84); and control again exceeded SMG on day 12. ORF 2155 followed the same course without the day-2 divergence. ORF 5692 behaved differently: both groups were low and indistinguishable until day 8, after which the control underwent an exceptionally large, transient induction on day 10 (C: 254.92 ± 33.01 vs. SMG: 14.59 ± 2.67), an order of magnitude above any other gene, and remained significantly above SMG on day 12 (C: 49.77 ± 5.01 vs. SMG: 16.30 ± 5.61).

**Figure 7:**
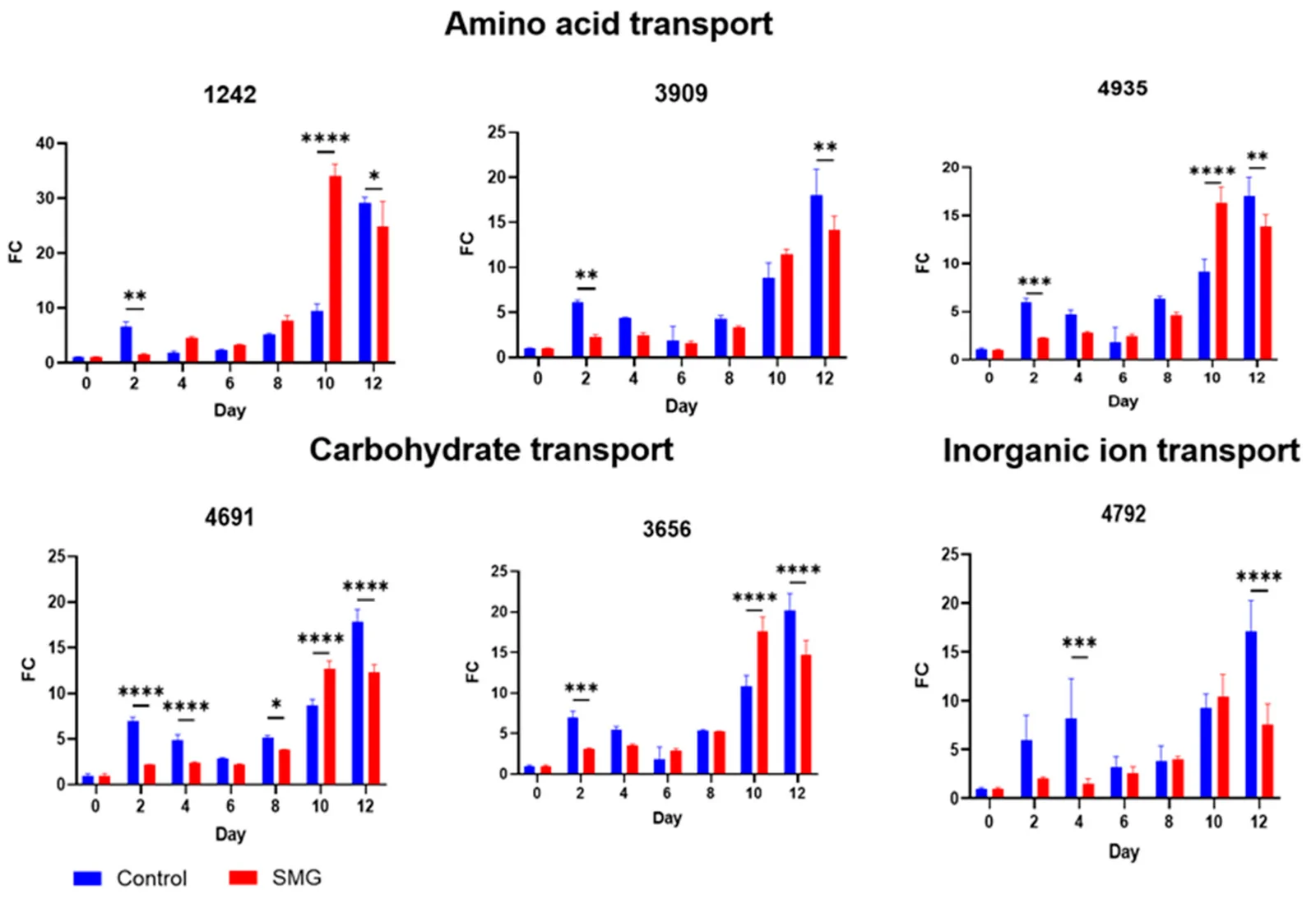
Transcript expression of functional open reading frames associated with amino acid transport, carbohydrate transport and inorganic ion transport in *L. platensis* in the control and SMG groups from day 0 to day 12, expressed as fold change (FC) relative to day 0 and normalized to the 16S rRNA gene. Data are mean ± SD of three independent biological replicates. **** *p* < 0.0001; *** *p* < 0.001; ** *p* < 0.01; * *p* < 0.05; control versus SMG at the same time point.

Among the open reading frames in Figure 7, ORF 1242, ORF 4935, ORF 4691 and ORF 3656 were significantly higher in the control on day 2, significantly higher under SMG on day 10 (most markedly ORF 1242, C: 9.47 ± 1.30 vs. SMG: 34.05 ± 2.16) and significantly higher in the control again on day 12. ORF 4691 was also higher in the control on day 4 and slightly higher on day 8. ORF 3909 was significantly higher in the control on day 2 and day 12, with a non-significant SMG excess on day 10, whereas ORF 4792 differed significantly only on day 4 and day 12 (C: 17.14 ± 3.12 vs. SMG: 7.55 ± 2.13).

### 3.3. Transcript Expression Compared at Matched Cell Density

Because the two cultures progressed through their growth curves with different timing, transcript abundance was also compared at time points with statistically indistinguishable cell densities: control day 6 (OD_750_ 0.846 ± 0.029) versus SMG day 10 (0.800 ± 0.062; *p* = 0.52), and control day 6 versus SMG day 12 (0.878 ± 0.017; *p* = 0.36). In the first comparison, 19 of the 20 genes were significantly more highly expressed under SMG, by 2.7- to 16.0-fold (Table S4): *ftsZ* 3.6-fold (*p* = 0.0002), photosystem I genes 4.0- to 5.8-fold, photosystem II genes 3.9- to 5.6-fold and open reading frames 3.5- to 16.0-fold; only ORF 5692, which was highly variable in the control, did not reach significance. The second comparison gave a consistent result (17 of 20 genes). The transcriptional divergence therefore cannot be attributed solely to growth stage, although density matching leaves the groups at different culture ages, so that density- and time-matched comparisons bracket, rather than fully resolve, the contributions of SMG and physiological state.

## 4. Discussion

Clinostat-based simulated microgravity is widely used to study responses to reduced directional gravitational cues and the associated mechanical forces [2,16,31], and *L. platensis* is of particular interest because of its role in bioregenerative life support and its nutritional value for spaceflight [6,11]. Here, control cultures appeared to proliferate faster initially but declined sharply after day 8 and died by day 14, whereas SMG cultures grew more slowly at first and sustained exponential growth to a final biomass 28.7% above the control maximum. The early lag under SMG is consistent with an adaptive burden: clinostat rotation continuously randomizes the gradients of nutrients, gases and light that sedimentation and convection establish under standard cultivation [13,15], altering mass transfer and self-shading [2,26]. Because the specific growth rate over days 0–6 did not differ (*p* = 0.13), the lag reflects delayed entry into exponential growth rather than suppressed growth capacity, and the main benefit of SMG is extended culture longevity rather than faster growth.

The control was motion-matched rather than growth-optimized: sealed 5 mL tubes on a rotator isolate the effect of gravity randomization but are not designed for prolonged batch cultivation. Three factors plausibly terminated the control culture by day 14. First, the small volume and headspace limit gas exchange, so a fast-growing culture depletes inorganic carbon and accumulates oxygen sooner than a sparged photobioreactor would. Second, the unreplenished Zarrouk medium was exhausted earlier by the faster-growing control than by the SMG culture; nutrient limitation in this organism shifts biomass from protein towards carbohydrate and ultimately causes pigment degradation and lysis [26,28], a recognized limit of batch cultivation that continuous or medium-recycling systems are designed to overcome [36]. Third, self-shading at high cell density compounds these effects [15]. Because residual nutrient concentrations were not measured, the relative contributions of nutrient depletion, carbon limitation and mechanical stress cannot be apportioned from these data.

A plausible explanation for the extended viability of the SMG culture is the reduction in mechanical shear stress [37]. In conventional cultures, cumulative hydrodynamic stress combined with nutrient depletion and the accumulation of metabolic byproducts precipitates a rapid culture crash [13,24]; the gentle, continuously reorienting motion of the clinostat may lower the shear acting on the filamentous trichomes and preserve membrane integrity and physiological activity for longer. Consistent with this, control cells lost their green pigmentation and showed pale-yellow cytoplasm from day 10 onward, a hallmark of chlorophyll degradation and cell death, whereas SMG cells retained normal coloration and morphology, in line with stress-related changes described previously in this organism [24]; live/dead staining confirmed that SMG cultures retained more viable cells while control cultures deteriorated.

*ftsZ* encodes the tubulin-homologous protein that forms the Z-ring and is essential for prokaryotic cytokinesis, so its transcript abundance is a useful proxy for cell-division activity [38,39]. Its expression tended to be higher in the control early in culture (significantly on day 2), consistent with the faster apparent initial proliferation, and was significantly higher under SMG on day 12, in line with the sustained proliferation of SMG cultures and the decline of the controls. Because FtsZ abundance in *Anabaena* PCC 7120 is shaped by carbon metabolism [38], this provides a link between the carbon status of the culture, its division activity and the growth trajectories observed. Earlier spaceflight and analogue studies of *Arthrospira*/*Limnospira* focused on growth kinetics, pigment content and physiological robustness [6,19,40]; the present work extends this understanding to the transcriptional regulation of cell division.

Among the photosystem and open reading frame genes, 11 of 19 (*psaB*, *psaC*, *psbDII*, *psbE* and seven open reading frames) showed a consistent time-dependent sequence: higher expression in the control on day 2, under SMG on day 10 (13 of the 19 genes) and in the control again on day 12 (all 19 genes), whereas *psaA*, *psaD* and *psbA* differed significantly only on day 12. This sequence recurred across functionally distinct gene sets, although not for every gene, suggesting a coordinated temporal shift in gene expression rather than purely gene-specific effects. Previous studies of this organism in space contexts characterized photosynthetic performance mainly at the physiological or biochemical level [12,41,42,43], and gene-resolved, time-course transcriptional data of this kind have not been widely reported.

This pattern can be reconciled with the growth and viability data if the day-12 surge in control expression is interpreted as a stress response accompanying culture collapse rather than as a sign of health. Declining cultures often show transient bursts of transcription as cells compensate for deteriorating conditions or lose regulatory control [8,44], and the exceptionally large, transient induction of ORF 5692 in the control on day 10, as the culture entered its death phase, suggests a dysregulated stress response. Conversely, the higher expression of *psaB*, *psaC*, *psbB*, *psbDII* and *psbE* under SMG on day 10, when SMG cultures were still proliferating and controls were declining, indicates that the photosynthetic machinery remained transcriptionally active and supported continued carbon fixation and energy production [10,20]. Together with the higher *ftsZ* expression on day 12, this suggests that SMG maintains an integrated state in which energy generation and cell division continue to operate cooperatively, providing a molecular explanation for the extended growth and higher final biomass.

Because gene expression changes with growth phase, differences at fixed sampling times could reflect different growth stages rather than SMG itself. In the density-matched analysis (Section 3.3), 19 of 20 genes remained significantly higher under SMG (17 of 20 against SMG day 12), so the divergence persists when biomass is controlled for. However, density matching introduces a difference in culture age just as time matching introduces a difference in cell density; the two analyses are complementary bounds on the same effect, and a definitive separation would require chemostat-style cultivation at a fixed dilution rate or transcriptome-wide sampling across a matched density series.

The slower initial growth under SMG reproduces the response of *L. indica* in a dedicated low-shear SMG setup, where it was accompanied by downregulation of ribosomal proteins and nitrate transporters and upregulation of carboxysome and photosystem proteins [6]. Our late-phase photosystem response is consistent with that observation, and the time course explains why single endpoints may appear contradictory: the sign of the difference depends on when the culture is sampled. The absence of a first-order microgravity effect on oxygen production by *L. indica* in an actively mixed, sparged flight photobioreactor [14] is also compatible with our data, because that system maintains the gas and nutrient supply that our closed tubes lack; the benefit of a low-shear environment is therefore most relevant to small, passively mixed volumes of the kind used in compact flight hardware. The 28.7% biomass gain is modest relative to the several-fold differences achievable by optimizing light intensity and temperature [15], placing gravity randomization as a secondary rather than a dominant determinant of productivity.

This study has limitations. A ground-based clinostat randomizes the gravity vector but reproduces neither true weightlessness nor the space radiation environment, so it models rather than demonstrates the spaceflight response [16]. Residual nutrient concentrations were not measured, so their role in the collapse of the control is inferred. Transcripts were normalized to 16S rRNA, which is stable in other cyanobacteria [35] but has not been validated under SMG in *Limnospira*; a second reference gene such as *rnpB* would strengthen quantification. Only a targeted panel of 20 genes was analysed, and mRNA abundance is an imperfect proxy for protein abundance and activity. Finally, biomass was estimated from optical density alone; dry weight, pigment and protein determinations are needed to establish nutritional yield.

## 5. Conclusions

In conclusion, *L. platensis* under simulated microgravity grew more slowly at first but sustained growth through day 14, whereas motion-matched controls peaked on day 8 and died by day 14, so that the final SMG biomass exceeded the control maximum by 28.7%. Because early specific growth rates were indistinguishable, the effect of SMG lies in the duration of the productive phase rather than the rate of growth. *ftsZ* was significantly lower under SMG on day 2 and higher on day 12, and in 11 of 19 photosystem and open reading frame genes the difference from the control reversed direction twice (control was higher early; SMG was higher around day 10; control was higher again on day 12); because higher expression under SMG persisted at matched cell density, it cannot be explained by growth stage alone.

The novelty of this work lies in resolving the response of *L. platensis* to simulated microgravity as a time-ordered transcriptional programme rather than a single endpoint, and in showing that the benefit of a low-shear, gravity-randomized environment is the postponement of culture collapse. For bioregenerative life support, a culture that sustains photosystem transcription and cell division for longer delivers oxygen and edible biomass more reliably, so the duration of the productive phase, rather than peak growth rate, becomes the parameter to optimize when sizing and operating a photobioreactor. The results also suggest that the low-shear conditions of spaceflight may be less detrimental to *L. platensis* cultivation than sometimes assumed, and days 10–12 are identified as the most informative sampling window for flight experiments. Confirming these findings in true microgravity, extending them to protein and pigment yield and testing the combined action of microgravity and ionizing radiation are priorities for further work.

## Figures and Tables

**Table 1:** Specific growth rate (µ) of *L. platensis* under control and simulated microgravity (SMG) conditions, calculated from optical density at 750 nm. Values are mean ± SD of five independent biological replicates; differences between groups were analyzed by multiple unpaired *t*-tests, with *p*-values adjusted for multiple comparisons using the Holm–Šídák method. *p* < 0.05 was considered significant.

Interval	Control µ (Day^−1^)	SMG µ (Day^−1^)	*p*
Days 0–6 (shared exponential phase)	0.172 ± 0.012	0.162 ± 0.006	0.13 (ns)
Full exponential phase of each culture (control, days 0–8; SMG, days 0–14)	0.162 ± 0.026	0.112 ± 0.006	0.006
Days 8–12 (late phase)	−0.111 ± 0.008	0.025 ± 0.011	<0.000001

## Data Availability

The original contributions presented in this study are included in the article. Further inquiries can be directed to the corresponding author.

## Supplementary Materials

The following supporting information can be downloaded at https://www.mdpi.com/article/10.3390/cimb48101061/s1.

## Abbreviations

The following abbreviations are used in this manuscript:

BLSSs	Bioregenerative life-support systems
C	Control
ESA	European Space Agency
FC	Fold change
GRAS	Generally Recognized As Safe
ISS	International Space Station
JAXA	Japan Aerospace Exploration Agency
MELiSSA	The Micro-Ecological Life Support System Alternative
OD	Optical density (measured at 750 nm, OD_750_)
ORF	Open reading frame
PSI	Photosystem I
PSII	Photosystem II
RT-qPCR	Reverse transcription quantitative real-time polymerase chain reaction
SMG	Simulated microgravity
SD	Standard deviation
ns	Not significant (*p* > 0.05)
µ	Specific growth rate
PPFD	Photosynthetic photon flux density
NCBI	National Center for Biotechnology Information
