# Supplementary material for: Effects of Simulated Microgravity on Limnospira platensis Growth and Transcript Expression Related to Photosystem and Open Reading Frames
Source: Curr Issues Mol Biol. 2026 Oct 14;48(10):1061. doi: 10.3390/cimb48101061 (PMC13703770; doi:10.3390/cimb48101061)
Supplement: Supplementary file 1 [file cimb-48-01061-s001.zip › cimb-4534804-supplementary.pdf]

Supplementary Materials

Table S1

Table S1. Composition of the Zarrouk medium used for the cultivation of *Limnospira platensis*, with catalogue numbers and suppliers of all reagents.

Component	Concentration	Catalog No.	Supplier
**Stock 1 (2× concentrated, 500 mL)**			
$NaHCO_3$	16.8 g L^{-1}	GC104004-500g	Servicebio, Wuhan, China
K_2HPO_4	0.5 g L^{-1}	P8281-100G	Sigma-Aldrich, St. Louis, MO, USA
**Stock 2 (2× concentrated, 500 mL)**			
$NaNO_3$	2.5 g L^{-1}	S0524.1000	Duchefa Biochemie, Haarlem, The Netherlands
K_2SO_4	1.0 g L^{-1}	P0772-250G	Sigma-Aldrich, St. Louis, MO, USA
NaCl	1.0 g L^{-1}	GC102004-500g	Servicebio, Wuhan, China
$MgSO_4{\cdot}7H_2O$	0.2 g L^{-1}	GC101004-100g	Servicebio, Wuhan, China
$CaCl_2{\cdot}2H_2O$	0.04 g L^{-1}	C7902-500G	Sigma-Aldrich, St. Louis, MO, USA
$FeSO_4{\cdot}7H_2O$	0.01 g L^{-1}	F8633-250G	Sigma-Aldrich, St. Louis, MO, USA
$Na_2EDTA{\cdot}2H_2O$	0.08 g L^{-1}	E5134	Sigma-Aldrich, St. Louis, MO, USA
**A5 trace element solution (added at 1 mL L^{-1})**			
H_3BO_3	2.86 g L^{-1} *	B6768-500G	Sigma-Aldrich, St. Louis, MO, USA
$MnCl_2{\cdot}4H_2O$	1.81 g L^{-1} *	M5005-100G	Sigma-Aldrich, St. Louis, MO, USA
$ZnSO_4{\cdot}7H_2O$	0.222 g L^{-1} *	Z0251-100G	Sigma-Aldrich, St. Louis, MO, USA
$CuSO_4{\cdot}5H_2O$	0.079 g L^{-1} *	CDB0063	Bio Basic Inc., Markham, ON, Canada
MoO_3	0.015 g L^{-1} *	267856	Sigma-Aldrich, St. Louis, MO, USA

Stock 1 and Stock 2 were prepared as 2×-concentrated solutions and stored separately to prevent precipitation of carbonate and phosphate salts. The complete medium (1 L) was obtained by combining 500 mL of Stock 1, 500 mL of Stock 2, and 1 mL of A5 solution, giving the final concentrations listed above. The pH was adjusted to 9.0 ± 0.2 and the medium was sterilized by autoclaving at 121 °C for 15 min. The formulation follows Zarrouk (1966). * Concentration within the A5 stock solution; the A5 solution was added to the medium at 1 mL L^{-1}.

Table S2

Table S2. List of primers used for RT-qPCR analysis in *Limnospira platensis*. For each gene the upper row gives the forward primer and the lower row the reverse primer.

Category	Gene / ORF	Primer sequence (5′→3′)	T_m (°C)	Source
**Proliferation**	*ftsZ*	GCTAACCCCTTTCGCCAAAA	58.4	Designed in this study (AP011615)
		CCGGAGACCTCGCTATCAAT	60.5	
**PSI**	*psaA*	TTCATTTGGCTGAGTGGTGC	58.4	
		TACCGGAAGCTCGCCATAAT	58.4	
	psaB	CTGAAAGCATTGCCACCACT	58.4	
		CAACGCACCTTTGACCAGAA	58.4	
	psaC	TAAACCCGAACGCTGAGGAA	58.4	
		CAGTGTGTTCGCGCTTGT	56.1	
	psaD	TCTCAGGGAAAACGCCATCT	58.4	
		GACCAGCACCAAAGAACAGG	60.5	
**PSII**	*psbA*	ACTCACGACCCATGTAGCAA	58.4	
		CATCATCTCTGGTGCGGTTG	60.5	
	psbB	TTTCTTCACAGAGGCGGGAT	58.4	
		GATGCAGAAGGCTGCGAATT	58.4	
	psbDII	CTTTCACCGGTCCTATTGCG	58.4	
		TGTGGAAGGGGTTGAGAGTC	60.5	
	psbE	TCCTGGCGCTCTTGAGTAAA	58.4	
		ACTACTGGTAGACGTGCTTT	58.4	
**ORF**	ORF 4935	ACGGGAGTTGATGTTATTG	53	Huili et al. [1], modified
		CTTAGGTTCAGCGGGTTG	56.1	
	ORF 3909	CCCACAACTCGCCTATCA	56.1	
		CTTCCACCAACTTGACCC	56.1	
	ORF 4133	AAGCCTCAGCCACAGTTA	53.8	
		GATAGCGTGTTTCATTTCC	53	
	ORF 4139	ATACGAGCCAGGAAGAAC	53.8	
		CGCTAATGGGATTTGATA	49.3	
	ORF 4792	GGGAGGTTATGCTATGGG	56.1	
		GTGGCTGCGTAATGGTTG	56.1	
	ORF 5692	CGCCCGAGACTTACAACC	58.4	
		GTGGCAGCATTTCCCTCT	56.1	
	ORF 1751	ATTTCCTCCATTGGTAGCG	55.2	
		TTAGCCTGATCCGAGTGC	56.1	
	ORF 1242	TGCCTTAGCCTTTGTAGC	53.8	
		TTCGGTTTCTTCCCAGAT	51.6	
	ORF 2155	CACTACCACGAGGTTTCAATAC	57.4	
		GTCGCTAACCCACCATCA	53.8	
	ORF 4691	GCATTTCCGCAAAGCAGT	53.8	
		CAACCAATTCGGGAAGGA	53.8	
	ORF 3656	AACCTATCCTCATATCCCTG	56.4	
		CCATCTTCCTTCTATCACCC	53.8	
	16S rRNA	AAGCCTGACGGAGCAAGA	58.4	Designed in this study (AP011615)
		GGACGCTTTACGCCCAAT	59.4	

PSI, photosystem I; PSII, photosystem II; ORF, open reading frame. The 16S rRNA gene was used as the reference gene for normalization. ORF numbering and the primers for the ORFs follow Huili et al. [1] (modified primers are indicated in the Source column); primers for *ftsZ*, the *psa* and *psb* genes and the 16S rRNA gene were designed against the *Arthrospira platensis* NIES-39 genome (GenBank accession AP011615).

Table S3

Table S3. Open reading frames (ORFs) analysed by RT-qPCR in *Limnospira platensis*. ORF numbers, protein accession numbers, gene products and functional categories are taken from Huili et al. [33], matched organisms are those of the database entries reported by Huili et al. [1] (*L. platensis* str. Paraca; *L. maxima* CS-328).

ORF	Accession number	Gene product	Functional category (COG)	Matched organism
1242	ZP_06381042	Cysteine synthase A	Amino acid transport and metabolism	*L. platensis*
1751	ZP_06381424	NAD-dependent epimerase/dehydratase	Cell envelope biogenesis, outer membrane	*L. platensis*
2155	ZP_06382427	Phycobilisome rod-core linker polypeptide (CpcG)	Energy metabolism (photosynthesis, respiratory electron transport)	*L. platensis*
3656	ZP_06380758	Fructose-1,6-bisphosphate aldolase	Carbohydrate transport and metabolism	*L. platensis*
3909	ZP_06382239	Aspartate-semialdehyde dehydrogenase	Amino acid transport and metabolism	*L. platensis*
4133	ZP_03275242	Pyridoxal phosphate biosynthetic protein PdxJ	Coenzyme metabolism	*L. maxima*
4139	ZP_06381750	Tetrahydrofolate dehydrogenase/cyclohydrolase	Coenzyme metabolism	*L. platensis*
4691	ZP_06382051	Transketolase	Carbohydrate transport and metabolism	*L. platensis*
4792	ZP_06382815	DNA starvation/stationary phase protection protein (Dps)	Inorganic ion transport and metabolism	*L. platensis*
4935	ZP_06381012	Acetolactate synthase, regulatory subunit	Amino acid transport and metabolism	*L. platensis*
5692	ZP_06383693	Phosphate uridyltransferase/glucosamine-phosphate acetyltransferase	Cell envelope biogenesis, outer membrane	*L. platensis*

COG, Clusters of Orthologous Groups; ORF, open reading frame.

Table S4

1. Optical density at 750 nm (OD_{750})

Control group

Day 0	Day 2	Day 4	Day 6	Day 8	Day 10	Day 12	Day 14
0.3	0.34	0.74	0.79	0.95	0.61	0.72	
0.3	0.36	0.58	0.90	1.02	0.62	0.74	
0.3	0.38	0.58	0.81	1.59	0.65	0.68	DEAD
0.3	0.40	0.50	0.80	1.00	0.61	0.72	
0.3	0.34	0.59	0.93	1.01	0.62	0.72	

SMG group

Day 0	Day 2	Day 4	Day 6	Day 8	Day 10	Day 12	Day 14
0.3	0.32	0.48	0.79	0.73	0.78	0.82	1.35
0.3	0.33	0.47	0.83	0.83	0.75	0.92	1.52
0.3	0.36	0.43	0.81	0.82	0.78	0.87	1.48
0.3	0.32	0.46	0.78	0.85	1.03	0.89	1.55
0.3	0.34	0.43	0.76	0.74	0.66	0.89	1.27

2. Cycle threshold (Ct) values of the target genes

2.1. Reference

16S

	day 0	day 2	day 4	day 6	day 8	day 10	day 12		day 0	day 2	day 4	day 6	day 8	day 10	day 12
C	15.27	15.55	17.42	16.71	16.25	16.25	16.25	SMG	15.27	15.01	14.38	14.07	15.45	15.45	15.45
	15.5	15.69	17.27	16.71	15.94	15.94	15.94		15.5	14.74	15.06	14.46	14.49	14.49	14.49
	15.52	15.75	17.39	16.26	15.96	15.96	15.96		15.52	14.47	14.27	13.71	14.22	14.22	14.22

ftsZ

	day 0	day 2	day 4	day 6	day 8	day 10	day 12		day 0	day 2	day 4	day 6	day 8	day 10	day 12
C	24.26	21.28	23.78	23.55	22.12	20.99	21.35	SMG	24.26	21.79	21.97	21.16	21.07	20.07	19.36
	23.62	21.08	23.37	23.11	22.06	21.07	20.59		23.62	21.69	21.37	21.13	20.8	19.33	18.99
	23.2	20.65	23.1	22.87	22.13	21.03	20.19		23.2	21.68	21.43	21.24	20.95	19.08	18.46

2.2. Photosystem I

psaA

	day 0	day 2	day 4	day 6	day 8	day 10	day 12		day 0	day 2	day 4	day 6	day 8	day 10	day 12
C	23.43	20.7	22.27	22.42	21.02	20.63	19.28	SMG	23.43	21.01	20.48	20.24	19.7	18.23	17.96
	22.72	20.3	22.21	22.03	20.59	19.88	18.67		22.72	20.53	20.12	20.1	19.7	17.98	17.63
	22.48	20.12	22.11	21.66	20.46	19.42	18.56		22.48	20.32	19.92	19.68	19.6	17.97	19.38

psaB

	day 0	day 2	day 4	day 6	day 8	day 10	day 12		day 0	day 2	day 4	day 6	day 8	day 10	day 12
C	24.98	21.52	24.09	23.27	22.37	21.61	20.13	SMG	24.98	22.10	22.06	22.17	21.28	19.79	19.11
	23.98	21.22	23.62	23.35	22.28	21.39	19.94		23.98	21.70	21.57	21.25	21.13	19.34	19.23
	23.31	21.05	23.33	23.11	21.95	21.18	20.05		23.31	21.66	21.46	20.94	20.93	19.11	19.16

psaC

	day 0	day 2	day 4	day 6	day 8	day 10	day 12		day 0	day 2	day 4	day 6	day 8	day 10	day 12
C	26.24	23.72	NaN	25.86	24.64	23.65	22.21	SMG	26.24	24.21	23.89	23.26	23.54	21.37	21.79
	26.02	23.74	25.67	25.46	24.22	23.24	22.56		26.02	24.08	23.64	23.25	22.74	21.18	21.21
	25.51	23.50	25.37	25.48	24.07	23.27	22.38		25.51	24.05	23.53	22.98	22.49	21.29	21.37

psaD

	day 0	day 2	day 4	day 6	day 8	day 10	day 12		day 0	day 2	day 4	day 6	day 8	day 10	day 12
C	22.82	20.07	22.51	22.25	21.12	20.07	19.12	SMG	22.82	20.78	20.72	20.47	20.01	18.70	18.73
	22.78	20.27	22.49	22.19	20.91	19.75	18.95		22.78	20.54	20.43	20.37	19.89	18.26	18.03

	22.55	20.30	22.36	22.10	20.59	20.53	18.59		22.55	20.37	20.25	19.97	19.82	17.85	18.16

2.3. Photosystem II

psbA

	day 0	day 2	day 4	day 6	day 8	day 10	day 12		day 0	day 2	day 4	day 6	day 8	day 10	day 12
C	22.10	20.13	22.38	22.54	21.28	20.12	19.11	SMG	22.10	20.76	21.21	20.33	20.08	19.33	19.68
	22.15	20.24	22.39	22.07	20.89	19.82	18.62		22.15	20.58	20.30	20.02	19.56	18.04	18.37
	22.05	20.00	22.15	21.81	20.61	19.62	18.25		22.05	20.37	19.91	19.65	19.57	17.69	18.19

psbB

	day 0	day 2	day 4	day 6	day 8	day 10	day 12		day 0	day 2	day 4	day 6	day 8	day 10	day 12
C	23.72	21.58	23.90	23.99	22.55	21.31	20.21	SMG	23.72	22.28	21.86	21.80	21.40	19.30	19.41
	23.56	21.32	23.27	23.37	22.41	21.19	20.16		23.56	22.08	21.54	21.11	21.13	19.04	19.31
	23.37	20.73	22.86	22.66	21.73	20.83	19.57		23.37	21.21	21.09	20.63	19.98	18.65	18.83

psbDII

	day 0	day 2	day 4	day 6	day 8	day 10	day 12		day 0	day 2	day 4	day 6	day 8	day 10	day 12
C	24.72	22.2	24.6	24.7	23.7	22.39	21.29	SMG	24.7	23.3	22.5	22.6	22.2	20.64	21.02
	24.63	22.2	24.3	24.4	23.1	22.6	21.05		24.6	22.8	22.6	22.4	22	20.33	20.35
	24.19	22	24.2	24.2	23.2	22.68	20.84		24.2	22.3	22.7	22.3	22.1	20.5	20.39

psbE

	day 0	day 2	day 4	day 6	day 8	day 10	day 12		day 0	day 2	day 4	day 6	day 8	day 10	day 12
C	25.02	21.87	24.38	24.03	22.76	21.77	20.54	SMG	25.02	22.38	22.48	21.66	21.47	20.04	20.07
	24.27	21.72	23.89	23.86	22.40	21.55	20.30		24.27	22.43	22.15	21.69	21.32	19.77	20.03
	24.14	21.55	23.73	23.58	22.40	21.58	20.46		24.14	22.53	21.92	21.68	21.25	19.77	20.38

2.4. ORF

ORF 1242

	day 0	day 2	day 4	day 6	day 8	day 10	day 12		day 0	day 2	day 4	day 6	day 8	day 10	day 12
C	26.12	23.57	27.04	26.33	24.54	23.86	22.06	SMG	26.12	24.80	23.18	23.23	22.56	20.46	20.94
	26.38	23.94	27.40	26.17	24.44	23.47	21.98		26.38	25.16	23.25	23.20	22.44	20.35	20.64
		23.76	27.43	26.06	24.48	23.56	21.96			25.06	23.13	23.13	22.78	20.53	21.15

ORF 4792

	day 0	day 2	day 4	day 6	day 8	day 10	day 12		day 0	day 2	day 4	day 6	day 8	day 10	day 12
C	34.99	33.31	33.26	34.98	33.83	32.61	31.49	SMG	34.99	33.10	33.49	31.92	32.24	30.89	30.98
	34.76	32.63	34.81	33.96	33.07	32.19	31.20		34.76	33.20	33.07	32.64	32.11	30.54	31.24
	35.07	32.06	33.79	34.42	34.12	32.25	31.72		35.07	33.38	34.03	32.12	32.29	31.18	31.83

ORF 4935

	day 0	day 2	day 4	day 6	day 8	day 10	day 12		day 0	day 2	day 4	day 6	day 8	day 10	day 12
C	23.53	21.42	23.33	23.69	21.81	21.45	20.49	SMG	23.53	21.75	21.36	21.15	20.78	19.07	19.26
	23.79	21.57	23.56	23.46	21.76	21.16	20.32		23.79	21.78	21.33	21.04	20.62	18.77	19.01
	23.62	21.37	23.57	23.34	21.68	21.05	20.16		23.62	21.85	21.22	20.87	20.76	18.89	19.15

ORF 3909

	day 0	day 2	day 4	day 6	day 8	day 10	day 12		day 0	day 2	day 4	day 6	day 8	day 10	day 12
C	23.66	21.29	23.51	23.54	22.37	21.49	20.4	SMG	23.66	21.96	21.66	21.82	21.37	19.5	19.36
	23.76	21.28	23.49	23.37	22.22	21.16	20.14		23.76	21.82	21.53	21.76	21.25	19.39	19.04
	23.67	21.39	23.48	23.21	22.1	20.93	19.93		23.67	21.66	21.38	21.45	21.18	19.54	19.12

ORF 3656

C	day 0	day 2	day 4	day 6	day 8	day 10	day 12	SMG	day 0	day 2	day 4	day 6	day 8	day 10	day 12

	22.73	20.16	22.29	22.68	21.09	20.3	19.34		22.73	20.5	20.14	19.96	19.78	18.16	18.47
	23	20.46	22.5	22.47	21.12	20.01	19.18		23	20.61	20.28	20.14	19.81	17.89	18.13
	22.97	20.34	22.35	22.52	21.06	19.95	19.04		22.97	20.65	20.2	19.91	19.79	18.1	18.33

ORF 4139

	day 0	day 2	day 4	day 6	day 8	day 10	day 12		day 0	day 2	day 4	day 6	day 8	day 10	day 12
C	18.03	19.01	21.58	20.44	19.57	19.03	18.03	SMG	18.03	20.27	18.96	18.39	18.34	16.84	17.33
	18.19	19.19	21.56	20.68	19.65	18.99	18.25		18.19	20.19	18.73	17.80	18.28	16.48	17.15
	18.06	19.13	21.75	20.63	19.60	18.93	18.22		18.06	20.06	18.78	18.08	18.24	16.87	17.09

ORF 5692

	day 0	day 2	day 4	day 6	day 8	day 10	day 12		day 0	day 2	day 4	day 6	day 8	day 10	day 12
C	36.14	34.06	38.72	35.05	38.03	29.23	31.82	SMG	36.14	33.41	33.57	33.60	33.30	32.18	31.82
	37.76	34.96	36.55		35.74	29.14	31.56		37.76	33.54	32.53	34.52	33.30	31.81	31.54
	36.52	33.11	34.01	39.66	35.06	29.51	31.56		36.52	34.32	35.10	34.01	33.46	32.30	32.60

ORF 2155

	day 0	day 2	day 4	day 6	day 8	day 10	day 12		day 0	day 2	day 4	day 6	day 8	day 10	day 12
C	23.70	21.57	23.53	24.15	22.23	21.70	20.41	SMG	23.70	21.55	21.68	21.50	20.98	19.36	19.52
	23.82	23.80	23.73	23.93	22.13	21.43	20.25		23.82	21.92	21.41	21.52	20.87	19.15	19.17
	23.41	21.61	23.55	23.75	22.05	21.31	20.16		23.41	21.78	21.33	21.26	20.97	19.30	19.45

ORF 4133

	day 0	day 2	day 4	day 6	day 8	day 10	day 12		day 0	day 2	day 4	day 6	day 8	day 10	day 12
C	20.53	19.22	21.48	21.08	20.01	19.39	18.10	SMG	20.53	19.76	19.29	19.12	18.47	16.97	17.13
	20.36	19.11	21.05	21.05	19.74	19.17	17.94		20.36	19.85	19.23	19.24	18.57	16.66	17.55
	20.28	19.05	21.10	20.84	19.64	19.23	18.18		20.28	19.76	19.13	19.14	18.36	16.70	17.26

ORF 4691

	day 0	day 2	day 4	day 6	day 8	day 10	day 12		day 0	day 2	day 4	day 6	day 8	day 10	day 12
C	24.11	21.42	23.68	23.42	22.16	21.51	20.43	SMG	24.11	22.06	21.71	21.31	21.24	19.48	19.46
	24.01	21.26	23.57	23.50	22.05	21.30	20.22		24.01	22.03	21.72	21.40	21.21	19.41	19.51
	23.56	21.25	23.33	23.47	22.13	21.33	20.36		23.56	22.08	21.85	21.44	21.23	19.61	19.65

ORF 1751

	day 0	day 2	day 4	day 6	day 8	day 10	day 12		day 0	day 2	day 4	day 6	day 8	day 10	day 12
C	20.39	19.84	23.46	22.31	21.02	20.02	19.10	SMG	20.39	20.64	19.93	19.59	19.37	17.75	18.01
	20.06	19.93	23.03	21.84	20.74	19.93	18.78		20.06	20.71	20.04	19.99	19.53	17.52	18.08
	20.12	19.83	23.02	21.90	20.82	19.80	18.83		20.12	20.50	20.15	19.85	19.14	17.80	18.15
